# Efficacy of L‐arabinose in managing cucumber Fusarium wilt and the underlying mechanism of action

**DOI:** 10.1002/ps.8523

**Published:** 2024-11-06

**Authors:** Min Yu, Rohyanti Yuliana, Stephany Angelia Tumewu, WanXue Bao, Haruhisa Suga, Masafumi Shimizu

**Affiliations:** ^1^ The United Graduate School of Agricultural Science Gifu University Gifu Japan; ^2^ Faculty of Applied Biological Sciences Gifu University Gifu Japan; ^3^ iGCORE Gifu University Gifu Japan; ^4^ Life Science Research Center Gifu University Gifu Japan

**Keywords:** arabinose, cucumber, Fusarium wilt, *Fusarium oxysporum*, disease resistance

## Abstract

**BACKGROUND:**

Cucumber Fusarium wilt (CFW), triggered by *Fusarium oxysporum* f. *sp. cucumerinum*, leads to substantial yield reductions in global cucumber (*Cucumis sativus* L.) production. Common management strategies for CFW include soil fumigation, grafting, and crop rotation. However, these methods have limitations regarding safety and efficacy stability, necessitating the development of new, cost‐effective, and eco‐friendly control strategies. Our prior research demonstrated that L‐arabinose, an inexpensive and safe sugar commonly used in food and beverages, effectively suppressed bacterial wilt in tomatoes. This study explores the potential of L‐arabinose in managing CFW and investigates its mechanism of action.

**RESULTS:**

Soil applications of L‐arabinose, ranging from 0.00001 to 0.01%, effectively suppressed CFW. The most significant suppressive effect was observed at 0.01%, reducing the disease severity index by 67.5% compared to the control treatment. Microscopic examination of transverse root sections showed that pathogen hyphae colonized the epidermis but seldom penetrated the cortical layer of roots in L‐arabinose‐treated seedlings. In contrast, the entire root tissue of control seedlings was colonized by the pathogen. Quantitative real‐time PCR revealed a significant increase in the expression of defense‐related genes dependent on salicylic acid, jasmonic acid, and ethylene in L‐arabinose‐treated plants compared to control plants, 6 and 10 days post pathogen inoculation.

**CONCLUSION:**

This study demonstrated that soil application of L‐arabinose can effectively suppress CFW by priming root tissues for multiple defense signaling pathways. Therefore, L‐arabinose holds potential as a new fungicide for managing CFW. © 2024 The Author(s). *Pest Management Science* published by John Wiley & Sons Ltd on behalf of Society of Chemical Industry.

## Graphical abstract

Soil application of L‐arabinose effectively suppressed cucumber Fusarium wilt (CFW) by priming robust defense responses in roots, suggesting the potential future use of L‐arabinose as an eco‐friendly fungicide for CFW.

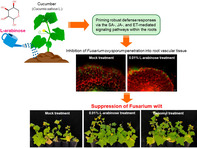

## 1 INTRODUCTION

*Fusarium oxysporum*, a widespread soil‐borne fungal pathogen, causes Fusarium wilt and damping‐off in over 100 plant species, leading to substantial agricultural losses worldwide.^1^, ^2^ As such, *F. oxysporum* ranks among the top 10 most harmful fungal phytopathogens.^3^ This pathogen invades root xylem tissue and spreads via the stem's vascular system, inhibiting nutrient and water transport and ultimately causing plant death.^4^ It can remain dormant and viable in soil for up to 30 years or more, even without a suitable host, by forming a durable resting structure known as a chlamydospore, making control extremely challenging.^5^

Cucumber (*Cucumis sativus* L.), a significant and popular vegetable, has an annual production value of approximately USD 9.8 billion worldwide.^6^ However, cucumber production is severely impacted by Fusarium wilt caused by F. oxysporum f. sp. cucumerinum. This disease, reported in many countries, can cause yield losses as high as 45%.^7^ Current management strategies include soil disinfestation with chemical fumigants,^8^ grafting seedlings onto resistant rootstocks,^9^ and crop rotation.^10^ However, each method has its limitations. Soil fumigants pose significant hazards to humans and the environment,^11^ necessitating reduced usage. Grafting seedlings is environmentally friendly and cost‐effective, but its effectiveness often diminishes due to the emergence of new pathogen races that overcome rootstock resistance.^12^ While crop rotation is an environmentally friendly and valuable strategy for reducing soil pathogen populations, its effectiveness is limited against soil‐borne pathogens that produce long‐lived structures like chlamydospores.^13^ These challenges underscore the need for new, cost‐effective, and safer strategies to control cucumber Fusarium wilt.

Natural products derived from plants, microorganisms, and other organisms have recently been recognized as eco‐friendly agents for disease control.^14^ These products are reported to protect plants from pathogen infection by either directly inhibiting pathogen growth or enhancing plant immunity. For instance, drenching roots with daphnetin, a plant‐derived hydroxycoumarin with bactericidal properties, has been shown to suppress the development of tobacco bacterial wilt caused by *Ralstonia pseudosolanacearum*.^15^ Similarly, applying sclareol, a labdane‐type diterpene extracted from tobacco, to roots has been found to inhibit bacterial wilt disease in tobacco, tomato, and *Arabidopsis* plants by activating multiple defense responses.^16^

In previous work, we reported that soil drenching with L‐arabinose, a major monosaccharide component in plant cell wall polysaccharides,^17^ could effectively suppress bacterial wilt in tomatoes.^17^ L‐arabinose is an aldopentose sugar containing five carbon atoms and including an aldehyde functional group and is a major monosaccharide component of plant cell wall polysaccharides.^18^ While the detailed mechanism of bacterial wilt suppression by L‐arabinose remains to be fully elucidated, it has been speculated that enhancing the immunity of tomato plants may be the main mode of action, as significant up‐regulation of a variety of defense‐related genes was observed in L‐arabinose‐treated tomato plants.^17^, ^19^ If L‐arabinose can enhance disease resistance in various plants, it could serve as a novel, environmentally friendly, and safe fungicide effective against multiple plant pathogens. Therefore, this study aimed to investigate the potential of L‐arabinose in controlling Fusarium wilt in cucumber plants and to explore its mode of action.

## 2 MATERIALS AND METHODS

### 2.1 Pathogen inoculum

In this study, *Fusarium oxysporum* f. sp. cucumerinum strain GUS77 was employed as the test pathogen. For fluorescence microscopy, we utilized FocuGFP‐10, a green fluorescent protein (GFP)‐tagged variant of GUS77. For the antifungal activity assessment, GUS77 was cultured on potato sucrose agar (PSA), composed of 200 g of potato, 200 g of sucrose, and 150 g of agar per 1 L deionized water, at 25 °C in darkness, serving as the inoculum. For plant inoculation experiments, a mycelial plug from a 3‐day‐old PSA culture plate was inoculated into potato sucrose broth, composed of 200 g of potato and 200 g of sucrose per 1 L deionized water, and incubated at 25 °C with shaking at 120 rpm for 4 days. The culture broth was then filtered to remove hyphae, and the filtrate was centrifuged at 2041 *x g* for 10 min. The resulting spores were suspended in 30 mL of sterile distilled water (SDW), and the spore concentration was adjusted to 1 × 10^4^ spores mL^−1^ for inoculation using a hemocytometer.

### 2.2 Antifungal activity test

This experiment was conducted to test the antifungal activity of L‐arabinose against Fusarium wilt pathogen.

Czapek‐Dox medium containing L‐arabinose at final concentrations of 0.01, 0.001, 0.0001, and 0.00001% (w/v) as the sole carbon source was prepared. Filter‐sterilized L‐arabinose solution was aseptically added to Czapek‐Dox agar containing no sucrose at 50–60 °C immediately after autoclaving. These media were then poured into 9‐cm Petri dishes and allowed to solidify. Czapek‐Dox agar medium containing no sugar was used as a control. Mycelial plugs, each 5 mm in diameter, were extracted from the edge of a 3‐day‐old PSA culture of GUS77 using a cork borer and positioned in the center of each medium. These plates were incubated at 25 °C in darkness. The diameter of the colony (mm) was measured 7 days post‐incubation. Each treatment was replicated across eight plates.

### 2.3 Evaluation of the suppressive effect of L‐arabinose against Fusarium wilt of cucumber

A pot experiment was conducted to assess the efficacy of soil drenching with L‐arabinose in suppressing Fusarium wilt of cucumber plants. Cucumber seeds (*Cucumis sativus* L. cv. Tokiwa‐jibai) were sterilized by immersing in 70% (v/v) ethanol for 1 min and 2% (v/v) sodium hypochlorite for 5 min, followed by six washes with SDW. The sterilized seeds were placed on moist filter paper in a Petri dish and allowed to germinate at 25 °C in darkness for 3 days. The germinated seeds were sown in plastic trays (Bee pot Y‐49; Canelon Kako Co., Ltd., Honjo, Saitama, Japan) containing a sterilized soil mixture of commercial potting soil (Saika‐ichiban; Ibigawa Kogyo Co., Ltd., Ogaki, Gifu, Japan) and commercial decomposed granite soil (Sabatsuchi; Tachikawa Heiwa Nouen Co., Ltd., Tochigi, Japan) (at a ratio of 1:1, w/w), covered with sterilized vermiculite, and grown in a controlled environmental chamber at 25 °C with a 12‐h light/dark cycle for 14 days. The cucumber seedlings were then transplanted into 9‐cm vinyl pots with two layers: the bottom layer contained 150 g of sterilized soil mixture inoculated with 10 mL of GUS77 spore suspension, and the top layer contained 150 g of sterilized soil mixture. After transplanting, each pot was drenched with 30‐mL of L‐arabinose solution at concentrations of 0.00001, 0.0001, 0.001, and 0.01% (w/v). For the control and benomyl treatments, the same volume of SDW and 100 mg L^−1^ benomyl (Benate Hydrate; Sumitomo Chemical; Tokyo; Japan), respectively, were applied to the pots. The pots were maintained in a controlled environmental chamber (25 °C, 12‐h light/dark cycle) for 28 days. During this period, each pot was fertilized weekly with 10 mL of a 250‐fold diluted Hyponex solution (type 6–9‐5; Hyponex Japan, Osaka, Japan). Disease severity was scored on a scale of 0–4 according to Roberts *et al*.^20^ (0 = healthy, 1 = <25% of leaves wilted, 2 = 25–50% of leaves wilted, 3 = 50–75% of leaves wilted, 4 = 75–100% of leaves wilted). The disease severity index (DSI) was calculated according to the following formula: DSI = [∑(the number of diseased plants in each scale × disease scale)/(total number of plants investigated × the highest disease scale)] × 100. Each treatment included five plants, and the experiment was conducted in triplicate (designated as trials 1–3).

### 2.4 Comparison of the suppressive effect of L‐arabinose and other aldopentoses against cucumber Fusarium wilt

This experiment was conducted to investigate whether the suppressive effect against Fusarium wilt is specific to L‐arabinose or common to aldopentose sugars. Therefore, the suppressive activity of various aldopentose sugars, including D‐arabinose, D‐ribose, and D‐xylose, against Fusarium wilt in cucumber plants was compared with that of L‐arabinose. A 30‐mL aliquot of a 0.01% (w/v) solution of each sugar was applied to the cucumber seedlings in pots inoculated with GUS77, as outlined in the previous pot experiment. The pots were then incubated in a controlled environmental chamber at 25 °C with a 12‐h dark cycle. Each pot was fertilized weekly with 10 mL of a 250‐fold diluted Hyponex solution. After 28 days post‐inoculation (dpi), the seedlings were assessed for disease severity using the previously described scoring system. Each treatment included five plants and the entire experiment was conducted in triplicate (designated as trials 1–3).

### 2.5 Cucumber seedling bioassay

As outlined below, L‐arabinose suppressed Fusarium wilt in pot experiments using autoclaved soil mixtures. However, microbes contaminated in the soil mixture during cucumber seedling cultivation could have proliferated using L‐arabinose as a carbon source, contributing to disease suppression. Consequently, subsequent experiments were conducted to ascertain if L‐arabinose's disease‐suppressing effect is contingent on soil microbial activity.

A soil mixture (commercial potting soil/decomposed granite soil in a 1:1 ratio by weight (w/w)) was sterilized by autoclaving at 121°C for 60 min, twice with a 24 h interval. Six grams of this soil were placed in a sterile flat‐bottomed glass tube (3 cm in diameter and 12 cm in height), treated with 1 mL of a sterile 0.01% (w/v) L‐arabinose solution, and inoculated with 2 mL of GUS77 spore suspension. The soil was then covered with 2 g of double‐autoclaved vermiculite. In the control treatment, the same volume of SDW was applied to the soil instead of the L‐arabinose solution, and the soil was inoculated with the pathogen. Surface‐sterilized pregerminated cucumber seeds were planted in the vermiculite layer (one seed per tube), covered with a small amount of double‐autoclaved vermiculite, and fertilized with 2 mL of a sterilized 250‐fold diluted Hyponex solution. These tubes were capped and incubated in a controlled environmental chamber (25 °C, 12‐h light/12‐h dark cycle) for 28 days, with weekly fertilization using 1 mL of a sterile 250‐fold diluted Hyponex solution. Disease severity was assessed using the scale described by Nishioka *et al*.,^21^ where 0 = healthy plant, 1 = cotyledon leaf yellowing and/or vascular browning, 2 = hypocotyl browning, and 3 = seedling dead. The DSI was calculated using the formula: DSI = [∑ (the number of diseased plants in each scale × disease scale)/(total number of plants investigated × the highest disease scale)] × 100. Each treatment included five plants, and the experiment was conducted in triplicate (designated as trials 1–3).

### 2.6 Quantification of *Fusarium oxysporum*

In this study, cucumber seedlings were subjected to a 0.01% (w/v) L‐arabinose solution treatment and then inoculated with a GUS77 suspension, following the procedure outlined for the cucumber seedling bioassay. Seedlings treated with SDW and inoculated with GUS77 served as controls. The GUS77 population density colonizing the cucumber roots was ascertained using a dilution plating method on FoG1 medium.^22^ After 10 dpi, asymptomatic seedlings were carefully removed from the glass tube. The taproots (approximately 2 cm long) were excised from the seedlings, rinsed gently with water, surface‐sterilized with 70% (v/v) ethanol for 1 min, and air‐dried for 5 min. Following the measurement of fresh weight, each root sample was homogenized in 1 mL of SDW using a homogenizer (BioMasher II®, Nippi, Tokyo, Japan) and serially diluted. A 300‐μL aliquot of each dilution was spread on three FoG1 medium plates per dilution and incubated at 25 °C in darkness for 7 days. The GUS77 population density was calculated as the average of three plants and expressed as log CFU g^−1^ of fresh root weight. This experiment was conducted in triplicate.

### 2.7 Fluorescence microscopy of cucumber roots

In a subsequent experiment, FocuGFP‐10 was employed as a challenge pathogen. Cucumber seeds sown in both mock‐treated and L‐arabinose‐treated sterile soil in test‐tubes were inoculated with FocuGFP‐10, following a similar procedure to the seedling bioassay described above. After a 14‐day incubation period at 25 °C under a 12‐h light/12‐h dark cycle, seedlings were carefully uprooted and gently rinsed with SDW to remove any adhering soil particles. The taproots excised from the seedlings were embedded in 4% (w/v) agarose filled in a Styrofoam pith hole to immobilize the fungal hyphae on the root surface. These roots were then sectioned using a plant microtome (MTH‐1, NK System, Osaka, Japan) to prepare 30‐μm thick transverse sections. Transverse sections were prepared from two specific locations on the taproots: one approximately 5–10 mm posterior to the root tip (lower) and the other 5–10 mm below the crown (upper). These sections were stained with propidium iodide (20 μg mL^−1^) in the dark for 15 min and subsequently rinsed three times with SDW. The stained specimens were then mounted on a glass slide for observation. Observations were made using an all‐in‐one fluorescence microscope (BZ‐X810, Keyence, Osaka, Japan). The green fluorescence was detected using a GFP filter (excitation 470/40 nm, emission 525/50 nm, dichroic 495 nm; OP‐87763, Keyence), while the red fluorescence was detected using a TexasRed filter (excitation 560/40 nm, emission 630/75 nm, dichroic 585 nm; OP‐87765, Keyence).

### 2.8 Analysis of defense‐related gene expression in cucumber roots by qRT‐PCR

In this study, we aimed to determine whether the treatment of cucumber plants with L‐arabinose induces defense responses. We analyzed the expression of four defense marker genes (*PR‐1a*, *PAL*, *LOX*, and *GluB*) using quantitative real‐time PCR (qRT‐PCR). Cucumber seedlings were treated with SDW, 0.01% (w/v) L‐arabinose, and 0.01% (w/v) D‐arabinose solution, and then inoculated with GUS77, following the procedure described for the cucumber seedling bioassay. At 3, 6, and 10 dpi, we collected 50 mg of roots from three plants for each treatment. Total RNA was extracted using the RNeasy Plant Mini Kit (Qiagen, Tokyo, Japan), and cDNA was synthesized from 1 μg of total RNA using the ReverTra Ace® qPCR RT Master Mix with gDNA Remover (Toyobo, Osaka, Japan), following the manufacturer's instructions. The qRT‐PCR analysis was performed using the Thermal Cycler Dice Real‐Time System *II* (Takara, Shiga, Japan). The reaction mix, with a final volume of 10 μL, consisted of 3 μL of RNase‐free water, 5 μL of TB Green® Fast qPCR Mix (Takara), 1 μL of the cDNA template, and 0.5 μL of 10 mM of each forward and reverse gene‐specific primer. The gene‐specific primers used in the present study are listed in Table 1. The cycling program was set as follows: 95 °C for 30 s, followed by 40 cycles of 95 °C for 5 s and 60 °C for 30 s. The actin gene was used as an internal standard to normalize each cDNA sample and calculate the relative expression values using the 2 − ^∆∆Ct^ method.^26^ The qRT‐PCR was conducted once, with three biological replicates for each treatment and two technical repetitions for each replicate.

**Table 1:** qRT‐PCR analysis: primers utilized for evaluating the expression of defense‐related genes in cucumber

Target gene	Forward primer (5′–3′)	Reverse primer (5′–3′)	Amplicon length (bp)	References
*PR‐1a*	AACTCTGGCGGACCTTAC	TCAATATGGCCTTTGGTATAAG	289	Dong *et al*.^23^
*PAL*	TCCACTCAACTGGGGTTTGG	TCTTCCACCATCCGCTTGAC	75	Abkhoo *et al*.^24^
*LOX*	ACTCTTTGAGCATATGGTTGGC	CCAAGAGTAGCTAAGGCTCCA	112	Abkhoo *et al*.^24^
*GluB*	TCGAACAGGAGGAGGATCTG	TCCAGGCTTTCTCGGACTAC	148	Okon Levy *et al*.^25^
*Actin*	AGTATTGTTGGTCGTCCCCG	TCAGTGAGAAGAACTGGGTGTTC	224	Dong *et al*.^23^

### 2.9 Statistical analysis

Data from the antifungal activity test and gene expression analysis were analyzed by one‐way ANOVA followed by the Tukey HSD test (*P* < 0.05). The results of the pot experiment were analyzed using two‐way ANOVA to determine how the disease severity index was affected by the two factors, ‘treatment’ and ‘trial’, and then analyzed by the *post hoc* Tukey HSD test for multiple comparisons (*P* < 0.05). Data from the cucumber seedling bioassay were also analyzed by two‐way ANOVA followed by Student's *t*‐test (*P* < 0.05). Counts of *F. oxysporum* were converted to logarithmic values and analyzed by one‐way ANOVA followed by Student's *t*‐test (*P* < 0.05).

## 3 RESULTS

### 3.1 Antifungal activity of L‐arabinose against *F. oxysporum*

**Figure 1:**
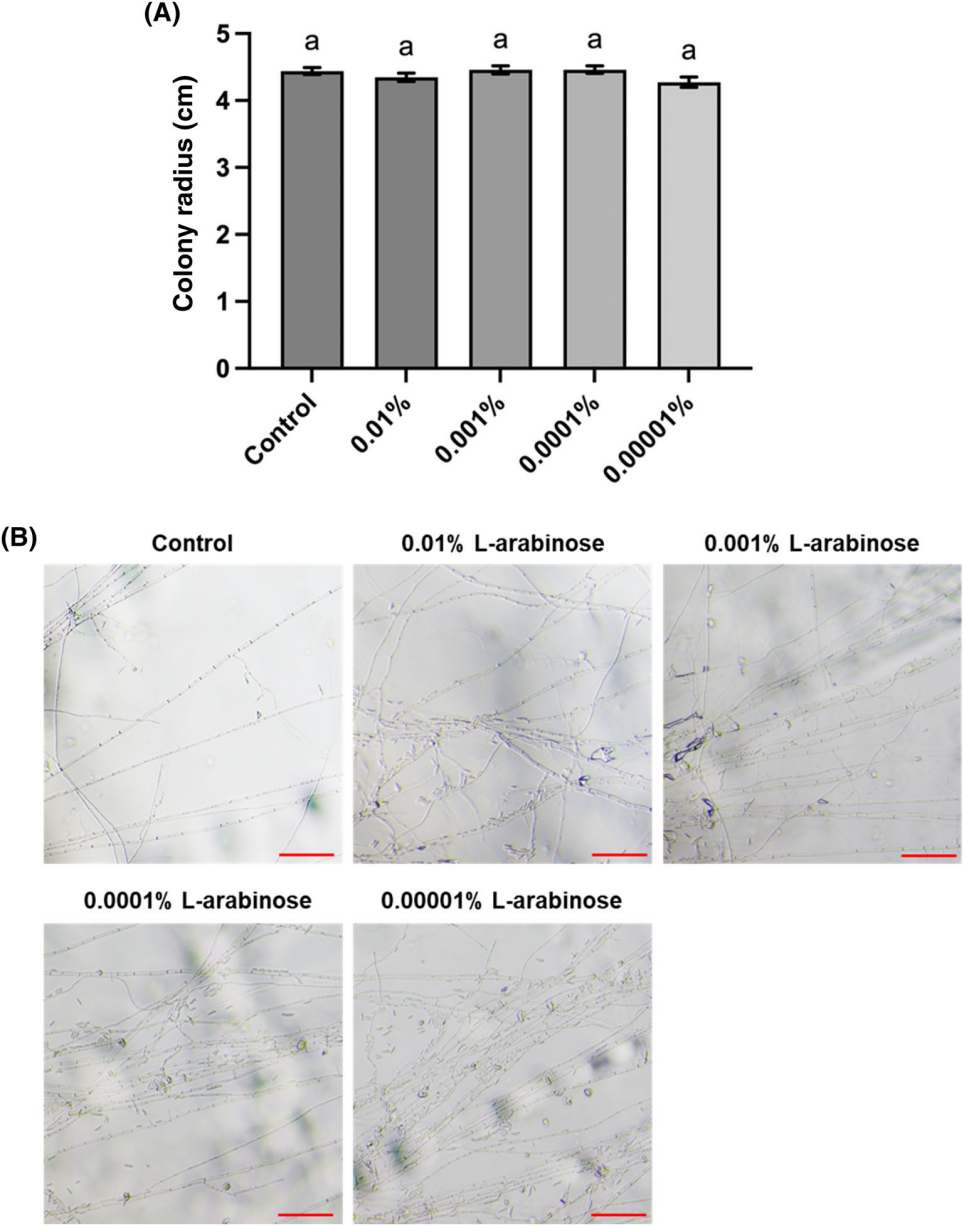
Influence of L‐arabinose on Fusarium oxysporum f. sp. cucumerinum growth. (A) Radius of F. cucumerinum f. sp. cucumerinum colony grown for 7 days on Czapek‐Dox agar medium without or with L‐arabinose as sole carbon source. The bars represent the mean ± standard deviation of eight replicates. Same letters above the bars indicate no significant difference between treatments (Tukey HSD test, *P* < 0.05). (B) Photomicrographs of hyphae from the leading edge of the fungal colony grown on each medium after 7 days of cultivation. Scale bar = 100 μm.

As shown in Fig. 1(A), the radial growth of the pathogen on Czapek‐Dox medium without sucrose was not inhibited by the addition of L‐arabinose at any of the concentrations tested. Microscopic observation of the hyphae from the leading edge of the colonies revealed that the growing hyphae of the pathogen on the L‐arabinose supplemented media showed no abnormality compared to those on the medium without L‐arabinose (Fig. 1(B)). These results suggest that L‐arabinose has no antifungal activity against this particular pathogen.

### 3.2 Suppressive effect of L‐arabinose against Fusarium wilt of cucumber

**Figure 2:**
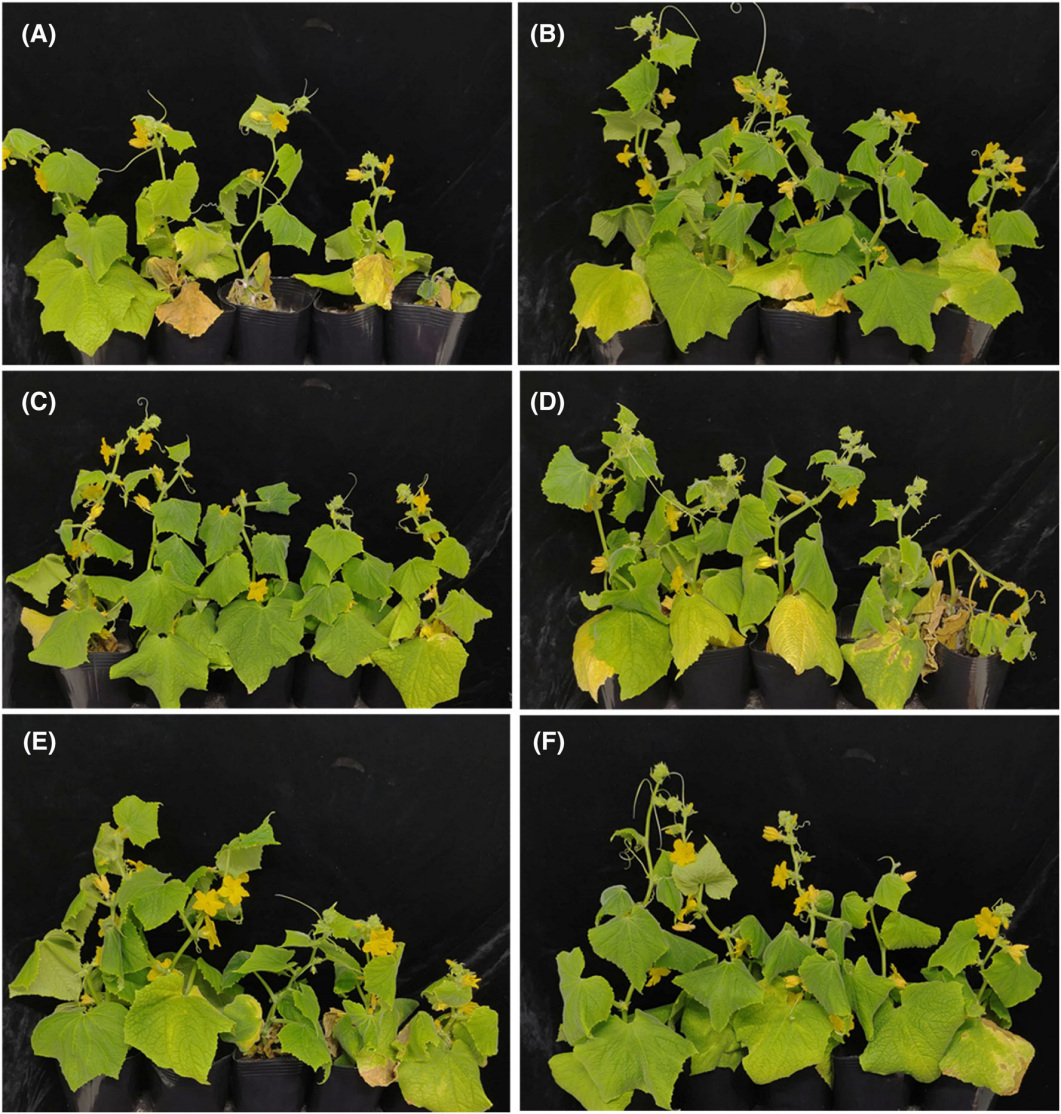
Visual comparison of Fusarium wilt symptoms in cucumber plants: effects of mock‐treatment *vs* L‐arabinose treatment. Cucumber plants, sown in soil inoculated with the pathogen, were drench‐treated with various solutions: (A) sterile distilled water, (B) benomyl, (C) 0.01% L‐arabinose, (D) 0.001% L‐arabinose, (E) 0.0001% L‐arabinose, and (F) 0.00001% L‐arabinose. Photographs were captured at 28 days post‐inoculation.

In the control treatment, many cucumber plants exhibited severe leaf chlorosis and eventually wilted (Fig. 2(A)). The mean DSI of the control plants reached 66.7% at 28 dpi (Table 2). Conversely, the development of symptoms in plants treated with benomyl was significantly suppressed, with the mean DSI reduced by approximately 42.6% compared to the control (Fig. 2(B) and Table 2). Plants treated with L‐arabinose displayed milder disease symptoms than control plants across all tested concentrations in all three trials (Fig. 2(C)–(F)). This observation indicates that L‐arabinose possesses activity that suppresses Fusarium wilt in cucumber plants. Notably, among the L‐arabinose treatments, the 0.01% L‐arabinose treatment demonstrated the most significant suppressive effect, reducing the mean DSI of three trials by 67.5%, a reduction comparable to that achieved with the benomyl treatment (Table 2).

**Table 2:** Impact of L‐arabinose application on Fusarium wilt disease severity in cucumber: observations from a pot experiment

Treatment	Disease severity index
Control (sterile distilled water)	66.7 ± 2.05a
100 mg L^‐1^ benomyl	38.3 ± 0.98ab
0.01% L‐arabinose	21.7 ± 2.05b
0.001% L‐arabinose	41.7 ± 2.77ab
0.0001% L‐arabinose	38.3 ± 2.42ab
0.00001% L‐arabinose	35.0 ± 2.45ab

*Note*: Disease severity index = [∑ (the number of diseased plants in each disease scale × disease scale)/(total number of plants investigated × the highest disease scale)] × 100%. Each value represents the mean ± standard deviation of three independent experiments. The results were analyzed by two‐way ANOVA followed by the Tukey HSD test. Two‐way ANOVA showed that only ‘treatment’ had a significant effect on the disease severity index. Different letters indicate significant differences between treatments (*P* < 0.05, Tukey HSD).

### 3.3 Suppressive effect of other aldopentoses against cucumber Fusarium wilt

**Figure 3:**
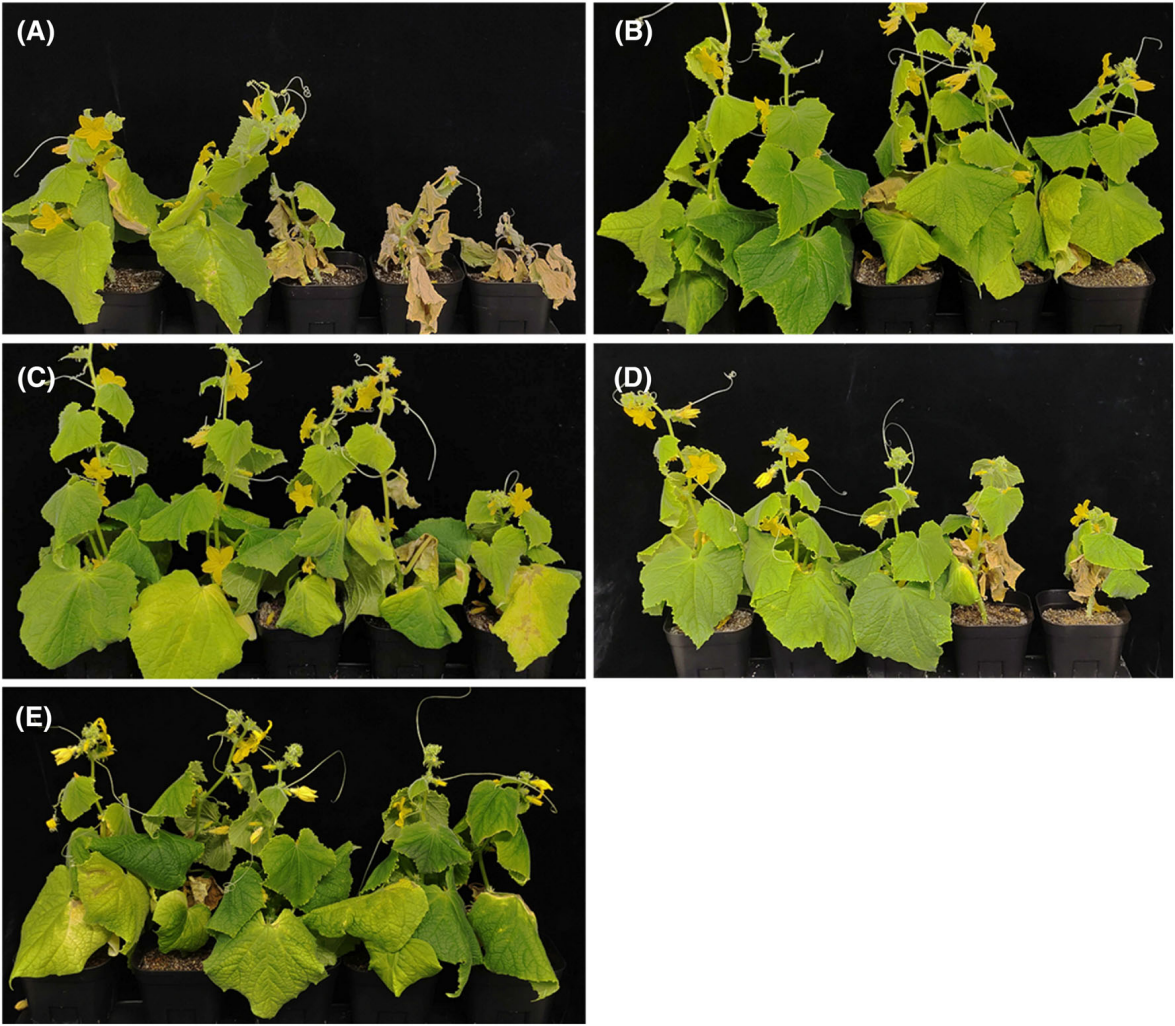
Comparative analysis of wilt symptoms in cucumber plants treated with L‐arabinose and other aldopentoses. Cucumber plants, sown in soil inoculated with the pathogen, were drench‐treated with various solutions: (A) sterile distilled water, (B) 0.01% L‐arabinose, (C) 0.01% D‐arabinose, (D) 0.01% D‐ribose, and (E) 0.01% D‐xylose. Photographs were taken at 28 days post‐inoculation.

In the control treatment, cucumber plants were heavily infected with the pathogen, resulting in a DSI of 68.3% at 28 dpi (Fig. 3(A) and Table 3). Conversely, plants treated with 0.01% L‐arabinose exhibited significantly suppressed symptom development, with a mean DSI of 21.7% (Fig. 3(B) and Table 3). Plants treated with other aldopentoses displayed slightly milder symptoms than the control plants in all three trials (Fig. 3(C)–(E)), leading to reduced mean DSI values ranging from 46.7% to 58.3% (Table 3). However, these treatments did not significantly differ from the control treatment in terms of mean DSI values (Table 3). These results suggest that while other aldopentoses may have some suppressive effects against Fusarium wilt, their efficacy is not as pronounced as that of L‐arabinose.

**Table 3:** Comparative analysis: effects of 0.01% L‐arabinose and other aldopentose applications on Fusarium wilt disease severity in cucumber in a pot experiment

Treatment	Disease severity index	
Control (sterile distilled water)	68.3 ± 2.05a
0.01% L‐arabinose	21.7 ± 1.86b
0.01% D‐arabinose	58.3 ± 1.39a
0.01% D‐ribose	48.3 ± 1.60a
0.01% D‐xylose	46.7 ± 0.98a

*Note*: Disease severity index = [∑ (the number of diseased plants in each disease scale × disease scale)/(total number of plants investigated × the highest disease scale)] × 100%. Each value represents the mean ± standard deviation of three independent experiments. The results were analyzed by two‐way ANOVA followed by the Tukey HSD test. Two‐way ANOVA showed that only ‘treatment’ had a significant effect on the disease severity index. Different letters indicate significant differences between treatments (*P* < 0.05, Tukey HSD).

### 3.4 Role of soil microbes in the wilt‐suppressive effect of L‐arabinose

**Figure 4:**
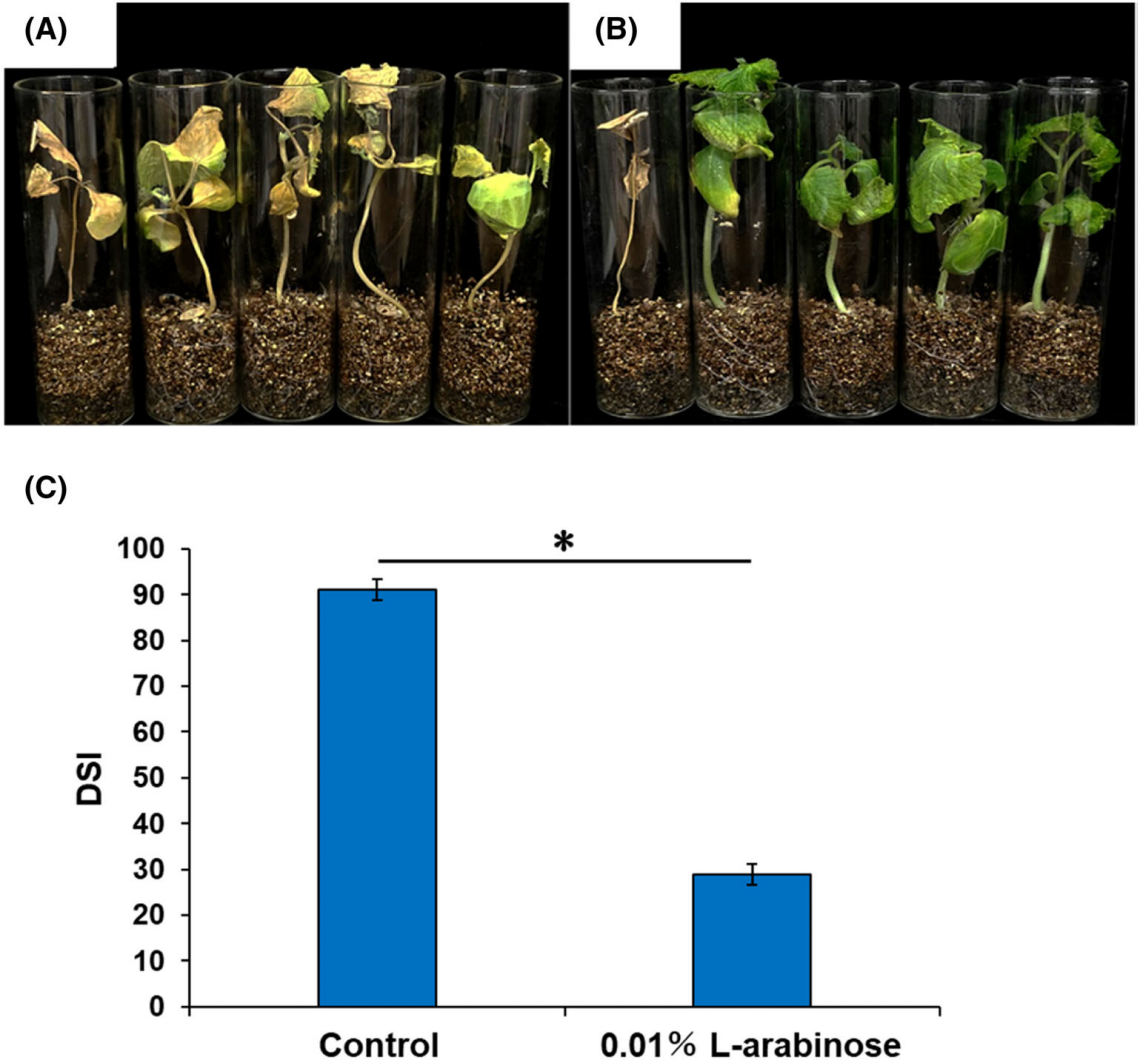
Suppression of Fusarium wilt in aseptically grown cucumber seedlings by L‐arabinose application. Fusarium wilt symptoms on (A) sterile distilled water (SDW)‐treated and (B) 0.01% L‐arabinose‐treated cucumber seedlings grown under aseptic conditions. Photographs were taken at 28 days post‐inoculation (dpi). (C) Disease severity index (DSI) of SDW‐treated (control) and L‐arabinose‐treated seedlings at 28 dpi. The bars represent the mean ± standard deviation of three independent experiments. The results were analyzed by two‐way ANOVA followed by the Tukey HSD test. Two‐way ANOVA showed that only ‘treatment’ had a significant effect on the disease severity index. The asterisk indicates a significant difference between treatments according to the Bonferroni *t*‐test at *P* < 0.05.

The effectiveness of L‐arabinose treatment was evaluated in a seedling bioassay conducted under aseptic conditions. Control seedlings were highly infected with the pathogen, with a mean DSI reaching 91.1% (Fig. 4(A),(C)). In contrast, L‐arabinose‐treated seedlings exhibited significantly suppressed pathogen infection compared to the control treatment in all three trials, reducing the mean DSI to 28.9% (Fig. 4(B),(C)). This result suggests that the suppression of Fusarium wilt by L‐arabinose treatment is not mediated by the activity of soil microbes.

### 3.5 Population density of *F. oxysporum* in cucumber roots

**Figure 5:**
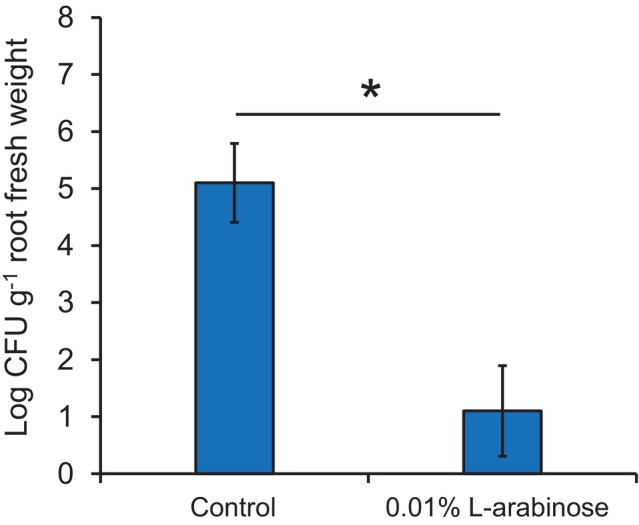
Comparison of *Fusarium oxysporum* populations in the roots of control and L‐arabinose‐treated cucumber plants. Population counts were conducted 10 days post‐inoculation. Homogenates from surface‐sterilized root samples were diluted and plated on FoG1 medium. The *F. oxysporum* colonies on the plates were counted after 7 days of incubation. The bars represent the mean ± standard deviation of three independent experiments. The asterisk denotes a significant difference between treatments according to the Student's *t*‐test at *P* < 0.05.

The population density of the pathogen invading the cucumber roots was compared between the control and L‐arabinose treatments at 10 dpi. In the control treatment, the pathogen population in the root tissue reached 5.1 log CFU g^−1^ of root fresh weight (Fig. 5). However, the population density of the pathogen in the roots of L‐arabinose‐treated plants was significantly lower, at 1.1 log CFU g^−1^ of root fresh weight. This result indicates that L‐arabinose either directly or indirectly suppresses the infection of the roots by the pathogen or the proliferation of the pathogen in the root tissue.

### 3.6 Effect of L‐arabinose treatment on *F. oxysporum* infection in cucumber roots

**Figure 6:**
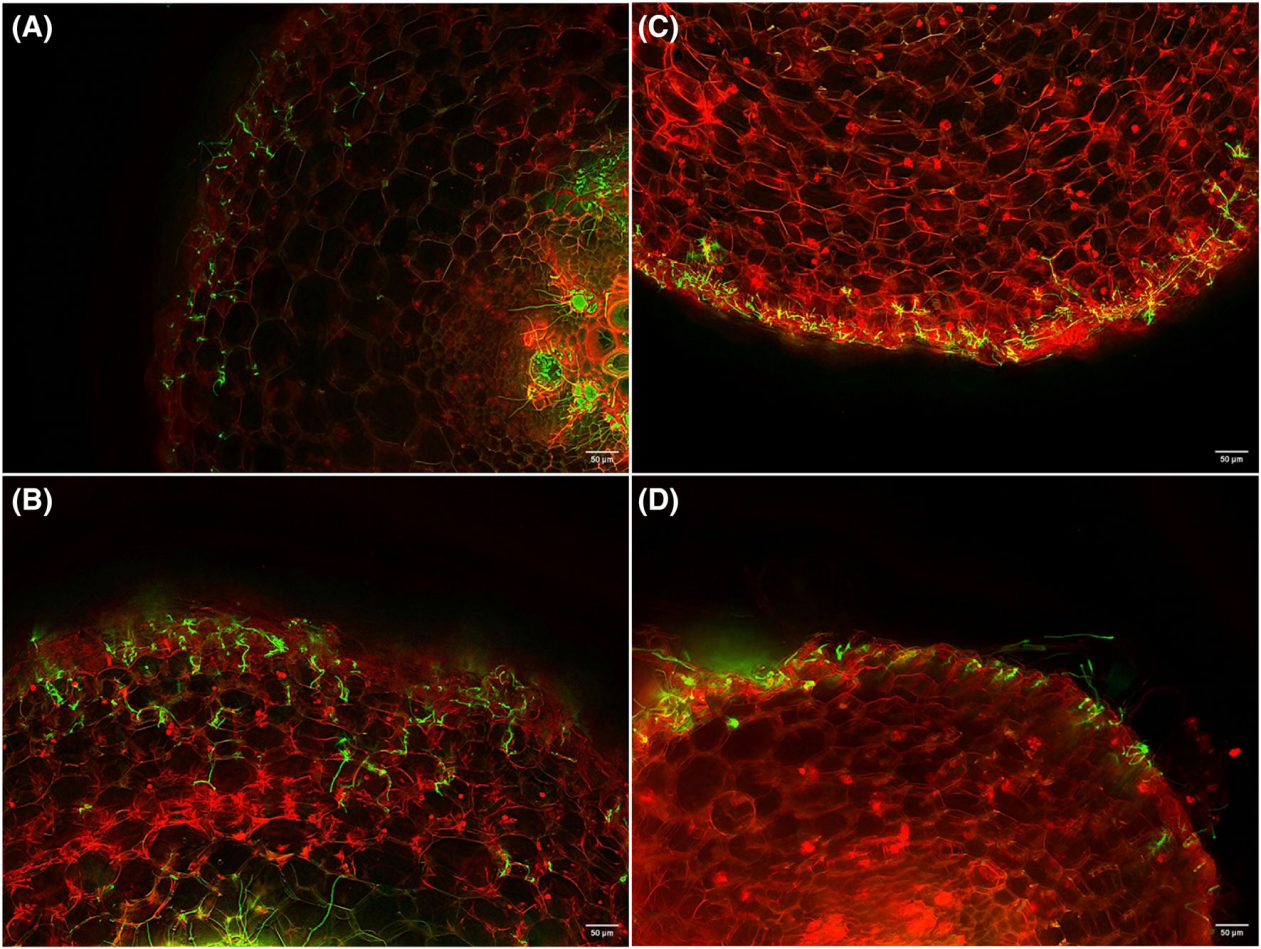
Visualization of cucumber root colonization by a green fluorescent protein‐tagged strain of Fusarium oxysporum f. sp. cucumerinum. Transverse sections from the upper position (5 to 10 mm below the crown) of roots of (A) control and (C) 0.01% L‐arabinose‐treated seedlings, and from the lower position (5–10 mm behind the root tip) of roots of (B) control and (D) 0.01% L‐arabinose‐treated seedlings. Sections were stained with propidium iodide to visualize the plant cell walls through red fluorescence. Observations were made at 14 days post‐inoculation. Scale bar = 50 μm.

To assess the impact of L‐arabinose treatment on pathogen infection in cucumber roots, we observed transverse sections of cucumber roots inoculated with the GFP‐tagged pathogen (FocuGFP‐10) using fluorescence microscopy at 14 dpi. In the control treatment, the pathogen's hyphae penetrated the epidermis and colonized the cortical layer, endodermis, and vascular tissue in both the lower and upper positions of the roots (Fig. 6(A),(B)). However, in the L‐arabinose treatment, while the pathogen proliferated on the surface and within the epidermis of the roots, similar to the control treatment (Fig. 6(C),(D)), the number of hyphae within the inner tissues was noticeably fewer than in the control plants.

### 3.7 Defense‐related gene expression in cucumber roots by qRT‐PCR

**Figure 7:**
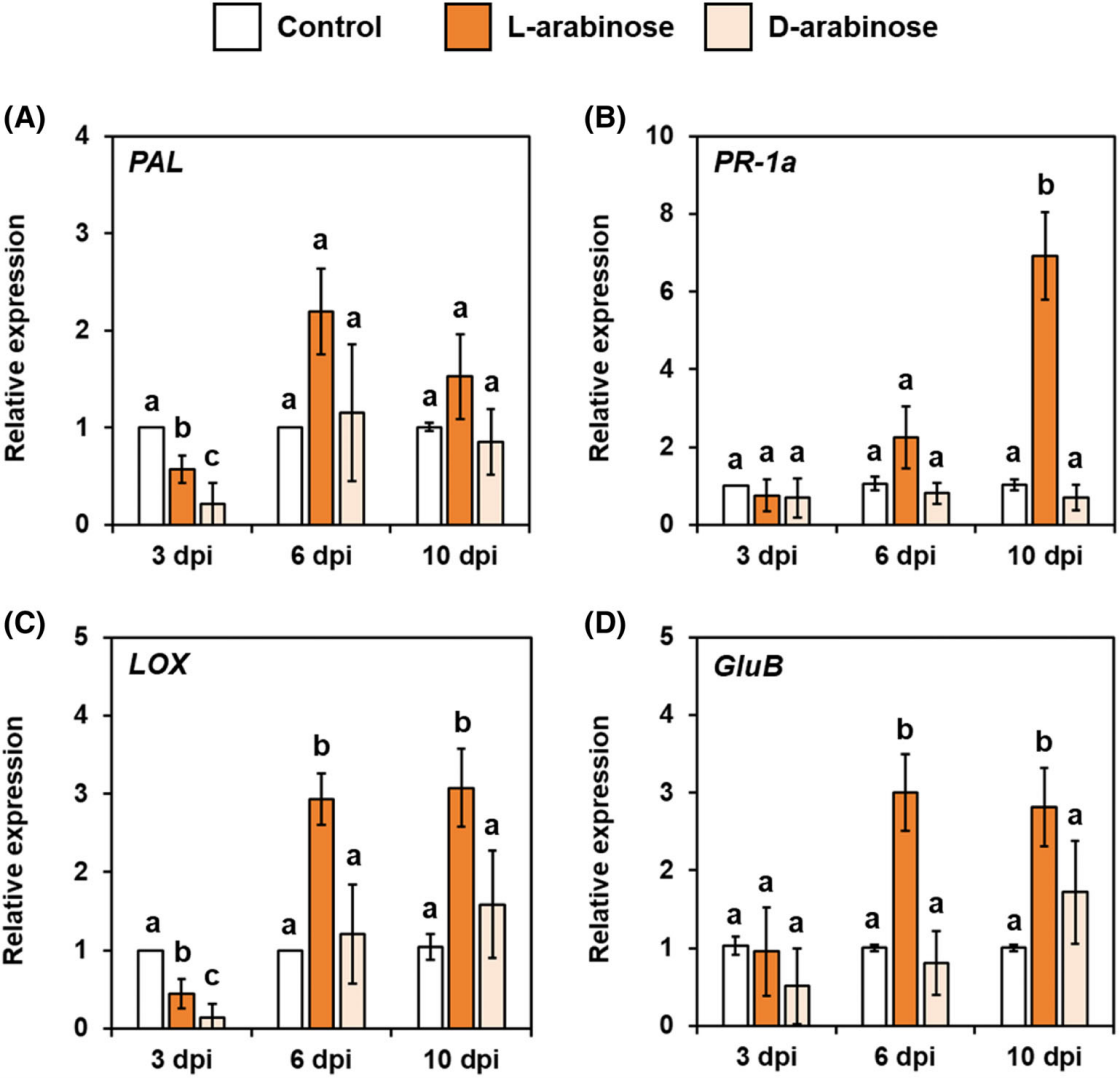
Relative expression of defense‐related genes in the roots of sterile distilled water‐, 0.01% L‐arabinose‐, and 0.01% D‐arabinose‐treated cucumber plants at 3, 6, and 10 days post‐inoculation (dpi). The genes include (A) *PAL*, (B) *PR‐1a*, (C) *LOX*, and (D) *GluB*. Expression was measured using quantitative real‐time PCR and normalized to the housekeeping gene *Actin*. Values represent the mean ± standard error in reference to the untreated control. Different letters indicate statistically significant differences according to Tukey's test at *P* < 0.05.

We also quantified the transcript levels of four defense‐related genes in both control and arabinose‐treated cucumber plants using qRT‐PCR at 3, 6, and 10 dpi. The application of L‐arabinose led to an increase in the relative expression of these genes in cucumber roots compared to the control, whereas D‐arabinose did not have the same effect. The expression of *PAL*, a gene that encodes a key enzyme for salicylic acid (SA) biosynthesis, was slightly up‐regulated in L‐arabinose‐treated plants compared to control and D‐arabinose‐treated plants at 6 and 10 dpi (Fig. 7(A)). The expression level of the SA‐responsive *PR‐1a* gene was also slightly elevated in L‐arabinose‐treated plants at 6 dpi and then significantly increased at 10 dpi compared to control and D‐arabinose‐treated plants (Fig. 7(B)). Furthermore, the expression of jasmonic acid (JA)‐responsive *LOX* and ethylene (ET)‐responsive *GluB* was significantly higher in L‐arabinose‐treated plants at 6 and 10 dpi than in control and D‐arabinose‐treated plants (Fig. 7(C),(D)). These results suggest that L‐arabinose treatment stimulates the SA‐, JA‐, and ET‐mediated defense mechanisms in cucumber plants.

## 4 DISCUSSION

As previously reported, the soil application of L‐arabinose has been effective in suppressing bacterial wilt in tomatoes.^17^ In this study, we investigated whether the application of L‐arabinose could also suppress Fusarium wilt in cucumbers. Our findings demonstrate that soil drenching with L‐arabinose does indeed have a suppressive effect on Fusarium wilt in cucumbers. Among the tested concentrations, a 0.01% L‐arabinose drench showed the highest efficacy, comparable to that of the chemical fungicide benomyl. This suppressive effect appears to be unique to L‐arabinose among natural aldopentoses, as other aldopentoses tested (i.e., D‐arabinose, D‐ribose, and D‐xylose) did not show significant activity. While the suppression of Fusarium wilt through the soil application of natural polysaccharides such as chitin, chitosan, peanut shell polysaccharides, and *Ganoderma lucidum* polysaccharides has been reported,^27^, ^28^, ^29^, ^30^ there has been no prior research on the management of Fusarium wilt through the soil application of natural simple sugars. To the best of our knowledge, this is the first study to demonstrate the suppressive effect of L‐arabinose on Fusarium wilt.

The spray application of D‐tagatose, a rare hexose monosaccharide, has been reported to control a broad spectrum of foliar diseases such as downy mildew and powdery mildew in various plants through its direct antifungal activity.^31^ According to the study, D‐tagatose was found to inhibit the hyphal growth of *Hyaloperonospora arabidopsidis*, the organism responsible for downy mildew in *Arabidopsis*, by interfering with the initial step of mannose metabolism. In another study by Chahed *et al*.,^32^ D‐tagatose was shown to inhibit the hyphal growth of *Phytophthora infestans*, the causal agent of late blight, primarily by disrupting mitochondrial processes. In contrast, L‐arabinose did not exhibit any *in vitro* antifungal activity against *F. oxysporum*. This finding suggests that L‐arabinose may suppress Fusarium wilt in cucumbers through mechanisms other than the direct inhibition of pathogen growth in the soil.

Many previous studies have indicated that amending soil with organic matter enhances soil microbial activity and community composition, thereby increasing the soil's suppressiveness to soil‐borne pathogens, including *F. oxysporum*.^33^, ^34^ For example, Nishioka *et al*.^35^ demonstrated that soil amendment with γ‐glutamyl‐S‐allylcysteine, a dipeptide synthesized by *Allium* plants, increased soil suppressiveness to Fusarium wilt by increasing the population of bacterial antagonists, such as the *Pseudomonas fluorescens* species complex. Similarly, Ali *et al*.^36^ showed that applying a garlic substrate suppressed Fusarium wilt in cucumber, potentially by increasing the diversity of antagonistic bacteria and fungi. However, our study found that soil drenching with L‐arabinose could effectively suppress Fusarium wilt of cucumber even under sterile conditions. This suggests that the suppression of Fusarium wilt was due to mechanisms other than the increased fungistatic activity of soil microorganisms. This finding aligns with our previous study, where L‐arabinose application reduced bacterial wilt in aseptically grown tomato seedlings.^17^ In that study, it was suggested that the enhancement of defense responses in tomato plants was a possible primary mechanism of bacterial wilt suppression by L‐arabinose application, as the expression of certain defense‐related genes was significantly up‐regulated in the L‐arabinose‐treated tomato seedlings. To determine whether the enhancement of defense responses in cucumber plants was involved in the suppression of Fusarium wilt by L‐arabinose application, we examined the colonization of cucumber roots by F. oxysporum f. sp. cucumerinum. We found that its population in the roots of L‐arabinose‐treated seedlings was significantly lower than that in the roots of control seedlings. The finding aligns with our previous study, where L‐arabinose treatment decreased pathogen population in the plant tissues.^17^ Furthermore, fluorescence microscopy revealed that while *F. oxysporum* hyphae proliferated on the surface of the roots of L‐arabinose‐treated seedlings and grew into the underlying epidermal layer, the number of hyphae colonizing the cortical layer was drastically reduced compared to the control. These results suggest that L‐arabinose activates potent defense responses in the cortical cells, thereby inhibiting the penetration of the Fusarium wilt pathogen into the vascular tissue. A range of physical and chemical defense responses have been identified as mechanisms that prevent root infection by the Fusarium wilt pathogen. Plant cultivars resistant to Fusarium wilt have been observed to halt the pathogen's progression in their roots by triggering multiple defense reactions. These include cell wall lignification, papilla formation in cells, synthesis of antifungal proteins, production of reactive oxygen species, and accumulation of phenolic compounds in the intercellular spaces of the cortical cell layer.^37^, ^38^ It has often been demonstrated that the artificial induction or priming of such defense responses, achieved by pretreating host plants with biological and chemical defense stimulants, can prevent infection by the pathogen, thereby effectively controlling Fusarium wilt.^39^, ^40^, ^41^, ^42^ For instance, pretreatment of carnation with the biocontrol bacterium *Pseudomonas* sp. strain WCS417r led to the synthesis and accumulation of phytoalexins, preventing systemic infection by *F. oxysporum* f. sp. *dianthi*.^43^ Generally, the three major phytohormones, SA, JA, and ET, are responsible for mediating defense responses to pests and pathogens.^44^ Individual or simultaneous activation of any of the SA‐, JA‐, or ET‐signaling pathways has been shown to enhance resistance to Fusarium wilt in several plants, including cucumber.^45^, ^46^, ^47^ In this study, we found that the expression of SA‐, JA‐, and ET‐responsive defense marker genes was strongly up‐regulated in the roots of L‐arabinose‐treated cucumber plants at 6 and 10 dpi, but not at 3 dpi. This suggests that L‐arabinose treatment primes cucumber roots for enhanced SA‐, JA‐, and ET‐mediated defense responses to attack by *F. oxysporum*, resulting in the inhibition of pathogen penetration into the cortical cell layers. This aligns with our previous study, where L‐arabinose treatment induced significant expression of SA‐ and ET‐responsive defense‐related genes in tomato plants following inoculation with the bacterial wilt pathogen.^17^ In addition, Fu *et al*. recently discovered through transcriptome analysis that the genes encoding key enzymes, such as cinnamyl alcohol dehydrogenase, for lignin biosynthesis were significantly up‐regulated in the roots and stems of L‐arabinose‐treated tomato plants, and concluded that the enhancement of cell wall physical strength may play an important role in the suppression of bacterial wilt by the application of L‐arabinose.^19^ Therefore, there is a possibility that similar responses are involved in the inhibition of the multiplication of Fusarium wilt pathogen in the roots of L‐arabinose‐treated cucumber plants. To understand the type of defense responses involved in the suppression of Fusarium wilt by L‐arabinose treatment, further detailed histochemical, biochemical, and molecular analyses are required.

Various molecules, including proteins, peptides, nucleotides, amino acids, and saccharides, which are derived from cells damaged by pathogens and herbivores, have been reported to act as danger signals. These are known as damage‐associated molecular patterns (DAMPs), and the exogenous application of these DAMPs triggers an immune response in plants.^48^ For example, the application of cell wall (CW)‐derived oligosaccharides has been found to induce resistance in plants to a wide array of plant pathogens, such as *Botrytis cinerea*, *H. arabidopsidis*, *Pseudomonas syringae*, and *Sclerotinia sclerotiorum*.^49^, ^50^ L‐arabinose, one of the common pentose sugars that constitute the hemicelluloses and pectin of plant cell walls,^51^ has been reported to be released from plant cell walls by the activity of arabinanase secreted by various fungal pathogens during the infection process.^52^, ^53^ Therefore, the application of L‐arabinose could mimic the enzymatic degradation of cell walls by the fungal pathogen, thereby activating DAMP‐triggered immunity in cucumber plants. CW‐derived DAMPs are presumably recognized at the cell surface by pattern recognition receptors (PRRs). However, only a few of the PRRs, such as the receptors for chitin and glucans, have been identified.^54^, ^55^ As reported in our previous and current studies, the application of monosaccharides other than L‐arabinose did not confer significant disease resistance in tomato and cucumber plants. This implies that various plants may possess a PRR that can specifically recognize L‐arabinose as a CW‐derived DAMP. This hypothesis warrants further investigation.

In summary, this study clearly demonstrated that the application of L‐arabinose to the soil effectively suppressed Fusarium wilt in cucumber plants. This was achieved by priming robust defense responses via multiple hormonal signaling pathways within the cucumber roots. L‐arabinose, a cost‐effective sugar widely used as a safe food and beverage additive, has significant potential for broad use as a new, environmentally friendly, and cost‐effective fungicide in the future. However, further research is required to investigate the detailed mechanism of L‐arabinose's action against *F. oxysporum* and to confirm the efficacy of L‐arabinose under field conditions.

## CONFLICT OF INTEREST

The authors declare that there are no conflicts of interest.

## Data Availability

Research data are not shared.
